# Unilateral Perforant Path Transection Does Not Alter Lateral Entorhinal Cortical or Hippocampal CA3 *Arc* Expression

**DOI:** 10.3389/fnsys.2022.920713

**Published:** 2022-06-30

**Authors:** Tara L. Cooper, John J. Thompson, Sean M. Turner, Cory Watson, Katelyn N. Lubke, Carly N. Logan, Andrew P. Maurer, Sara N. Burke

**Affiliations:** ^1^Department of Neuroscience, Evelyn F. McKnight Brain Institute, University of Florida, Gainesville, FL, United States; ^2^Graduate Program in Biomedical Sciences, Neuroscience Concentration, University of Florida, Gainesville, FL, United States

**Keywords:** entorhinal cortex, mnemonic similarity task, hippocampus, rat, immediate early gene

## Abstract

It is well established that degradation of perforant path fibers is associated with age-related cognitive dysfunction and CA3 hyperactivity. Whether this fiber loss triggers a cascade of other functional changes within the hippocampus circuit has not been causatively established, however. Thus, the current study evaluated the effect of perforant path fiber loss on neuronal activity in CA3 and layer II of the lateral entorhinal cortex (LEC) in relation to mnemonic similarity task performance. Expression of the immediate early gene *Arc* was quantified in rats that received a unilateral right hemisphere transection of the perforant path or sham surgery that cut the cortex but left the fibers intact. Behavior-related expression of *Arc* mRNA was measured to test the hypothesis that fiber loss leads to elevated activation of CA3 and LEC neurons, as previously observed in aged rats that were impaired on the mnemonic similarity task. Transection of perforant path fibers, which has previously been shown to lead to a decline in mnemonic similarity task performance, did not alter *Arc* expression. *Arc* expression in CA3, however, was correlated with task performance on the more difficult discrimination trials across both surgical groups. These observations further support a link between CA3 activity and mnemonic similarity task performance but suggest the reduced input from the entorhinal cortex to the hippocampus, as observed in old age, does not causatively elevate CA3 activity.

## Introduction

The perforant path is the primary fiber projection from the cortex to the hippocampus. A major component of this cortical to hippocampal white matter tract are the axons from layer II entorhinal cortical neurons that synapse in CA3 and the dentate gyrus (Amaral and Witter, 1995). This fiber projection is known to degrade in advanced age. This was first established by the observation that presynaptic fiber potential for a given stimulation intensity of perforant path axons was reduced in aged compared to young rats (Barnes and McNaughton, 1980; Foster et al., 1991). Later, stereological electron microscopy was used to show that in aged compared to young rats there were fewer synapses in the stratum lacunomsum of CA3 (Adams et al., 2010) and the dentate gyrus molecular layer (Geinisman et al., 1992). These dendritic regions of CA3 and dentate gyrus both receive direct input from layer II neurons in the entorhinal cortex. Age-related reductions in perforant path white matter integrity have also been shown in studies using magnetic resonance imaging in human study participants (Stoub et al., 2005; Yassa et al., 2010, 2011a; Rogalski et al., 2012; Bennett et al., 2015; Bennett and Stark, 2016).

While the number of neurons in the hippocampus (Rapp and Gallagher, 1996) and entorhinal cortex (Rapp et al., 2002) does not change with age, age-related deficits have been associated with hyperactivity in the CA3 subregion in rodents (Wilson et al., 2005; Robitsek et al., 2015; Lee H. et al., 2021), and non-human primates (Thomé et al., 2016). Additionally, fMRI studies in human study participants have reported that the BOLD signal in CA3/dentate gyrus is higher in older compared to younger adults (Yassa et al., 2010, 2011a; Reagh et al., 2018). This aberrant activity is hypothesized to underlie poor cognition and is exacerbated in persons with Mild Cognitive Impairment and Alzheimer’s disease (Bakker et al., 2012, 2015; Tran et al., 2017). While correlations between CA3 hyperactivity and poor cognitive outcomes in old age have been observed across species, the cellular changes that provoke this increased activity in aged animals is unknown.

One hypothesis is that perforant path fiber disruptions, discussed above, cause a reduction in the recruitment of interneuron activity and lead to an imbalance between inhibition and excitation. In support of this idea, CA3 hyperactivity correlates with the magnitude of perforant path degradation in humans (Yassa et al., 2011a,b). These data suggest that disruptions in this white matter tract could initiate a cascade of events that leads to CA3 hyperactivity and cognitive dysfunction. In support of this idea, aged rats that performed poorly on a rodent analog of the mnemonic similarity task (Stark et al., 2013; Johnson et al., 2017), showed a higher proportion of cells that were positive for mRNA of the immediate-early gene *Arc*, a neuronal marker for plasticity, in both CA3 and in the lateral entorhinal cortical (LEC) neurons that project to the hippocampus (Maurer et al., 2017). This observation suggests that a common underlying mechanism could be related to both LEC and CA3 dysfunction in cognitively impaired aged animals. Whether this is due to perforant path fiber disruption, however, has not been empirically examined.

Aged rodents (Burke et al., 2010; Johnson et al., 2017), monkeys (Burke et al., 2011; Johnson et al., 2017) and humans (Stark et al., 2013, 2015; Stark and Stark, 2017) are less accurate at the discrimination of similar stimuli that share features compared to young animals. Specifically, older study participants are more likely to classify novel lures as familiar when they share characteristics with a stimulus that has previously been experienced. This behavioral deficit can be recapitulated in young animals following a unilateral right hemisphere perforant path transection (Burke et al., 2018). This finding suggests that degradation of perforant path fibers in young animals may model cognitive deficits seen in aging that result in impaired behavior and discrimination ability. Potential changes in neuronal activity underlying behavioral deficits seen in young animals following disconnection of perforant path fibers and how these changes compare to data in aged animals showing CA3 hyperactivity have not been examined, however. Thus, the current study interrogated whether loss of perforant path fibers recapitulates CA3 hyperactivity seen in aged animals by quantifying the expression of the immediate-early gene *Arc* in relation to performance on the rodent mnemonic similarity task in rats that had received a unilateral perforant path transection.

In the current study, rats that received a right hemisphere perforant path transection and control animals (sham surgery that cut the cortex, leaving fibers intact) performed two epochs of the rodent mnemonic similarity task. In the different epochs, the amount of feature overlap of the two objects to be discriminated was either low (70%) or high (90%), and the order of epochs was counterbalanced across animals. *Arc* expression in relation to behavior was then examined in CA3 and cortical layer II of the LEC. The subcellular localization of the immediate-early gene *Arc* can be used to determine which neural ensembles across the brain were active during two distinct episodes of behavior. *Arc* is first transcribed within the nucleus of neurons 1 to 2 min after patterned synaptic activity associated with neuronal plasticity and cell firing. *Arc* mRNA then translocates to the cytoplasm approximately 15 to 20 min after cell firing, which allows for cellular activity during two epochs of behavior, separated by a 20-min rest to be represented within a single neural population (Guzowski et al., 1999). An observed increase in CA3 *Arc* expression following fiber transection would suggest a causative relationship between white matter damage and CA3 hyperactivity. Contrary to this hypothesis, perforant path fiber damage did not impact CA3 or LEC *Arc* expression suggesting that compensatory mechanisms were employed to normalize circuit activity in the face of fiber damage. Moreover, across both experimental groups there was not a difference in the population overlap between the LEC and CA3, which calls into question network models of pattern completion/separation computation in the hippocampus.

## Materials and Methods

### Animals

Experiments used 37 young (4–6 months old) male and female F344 x Brown Norway F1 hybrid rats (17 male/20 female) that were 4 months old at arrival to the UF vivarium. A total of 19 female rats (6 Cut/12 Sham) and 13 male rats (6 Cut/7 Sham) underwent surgical procedures, behavioral testing, and *Arc* catFISH analysis. The others were either positive controls to verify staining or negative controls to verify baseline Arc expression without behavioral experience. Rats were single-housed on a reversed 12-h light/dark cycle and experiments were conducted during the dark phase. When the rats arrived at the facility, they were given 7 days to acclimate prior to initiating food restriction. All rats were then handled for an additional 7 days prior to behavioral shaping. The behavioral training, testing procedures, and results are as described in Burke et al. (2018). Briefly, experimentation and training occurred at least 5 days per week at approximately the same time each day. Before experimentation, rats were placed on a restricted diet of a moist chow (20 ± 5 g; ∼39 kcal/day; Teklad LM-485, Harlan Labs) to provide a food-based motivation for behavioral experiments. Drinking water was given *ad libitum*. Once rats had reached 85% of their baseline body weight, they began habituation to the testing procedures. All animals handling procedures and protocols were carried out in accordance with NIH *Guide for the Care and Use of Laboratory Animals* and approved by the Institutional Animal Care and Use Committee at the University of Florida. Note that behavioral data collected from these same rats have been presented elsewhere (Burke et al., 2018). These data were collected from a variant of the mnemonic similarity that measured the extent to which feature overlap affects performance across four different target-lure overlap conditions. The behavioral conditions were distinct from those used here, which were designed specifically to compare *Arc* expression across two different overlap conditions. Aside from the verification of perforant path fiber transection, none of the current data have been presented previously.

### Apparatus

**FIGURE 1:**
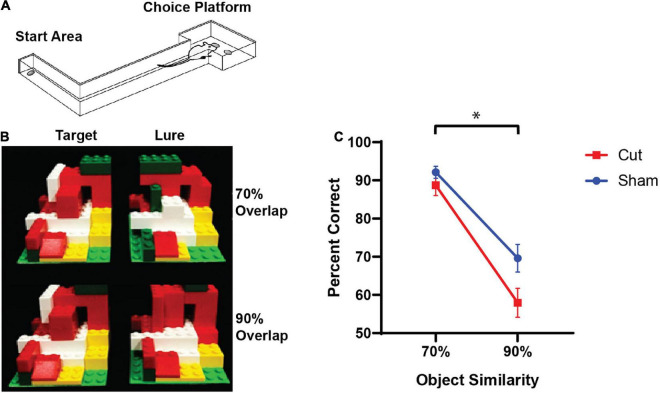
The rodent mnemonic discrimination task. **(A)** Schematic of the L-shaped apparatus used for both training and testing discrimination ability in rats. **(B)** The LEGO block object pairs used in testing. Rats were trained to identify the target object as correct compared to either a 70% (top) or 90% (bottom) visual overlap object. **(C)** Mean percent correct trials on target-lure discrimination task (n’s: Sham = 12♀/7♂; Cut = 12♀/7♂). Rats performed worse on the higher similarity lure trials (*F*_[1,28]_ = 62.361, *p* < 0.001). Furthermore, there was a main effect of experimental surgical condition (*F*_[1,28]_ = 6.072, *p* = 0.020), with Sham animals having more correct trials compared to the Cut rats. There was no main effect of sex on performance (*F*_[1,28]_ = 0.752, *p* = 0.393). Error bars are ± one standard error of the mean (SEM). *Indicates significant effect.

All experimentation and training procedures were conducted in an L-shaped track as shown in Figure 1A and previously described in Johnson et al. (2017). The track contained a starting area, narrow track measuring 84 cm × 10.2 cm with 6.4 cm walls on both sides, and a choice platform measuring 32 cm × 24 cm also with walls 6.4 cm in height. There were two food reward wells, each 2.5 cm in diameter, centered in the choice platform 12 cm apart, 7.6 cm from the back wall, and recessed 1 cm into the floor. All experiments took place in a dimly lit testing room with a red 60 W light bulb near the starting area and another shining on the choice platform. An additional white 60 W light bulb was placed in a lamp that aimed at the ceiling in the corner of the room to facilitate video monitoring and as an additional light source for illumination of the object stimuli used in the experiments and in previous experiments (Burke et al., 2018). A webcam (Logitech; Newark, CA, United States) was mounted 50 cm above the choice platform to produce video recordings of discrimination trials. Trials were recorded using custom software (Collector; Burke/Maurer, Gainesville, FL, United States). Additionally, a white noise machine was used during all testing session, in order to reduce the likelihood that external noise would cause distraction.

### Habituation and Shaping

Prior to the beginning of habituation, rats were given Froot Loops cereal pieces along with the rat chow, in order to familiarize the animals with the reward. At the beginning of habituation, rats were placed inside the L-shaped track near the starting area and explored the track for 10 min, during which they retrieved Froot Loops cereal pieces throughout the track (Kellogg’s; Battle Creek, MI, United States). Habituation usually lasted anywhere from 1 to 3 days and shaping began when rats could explore the complete L-shaped track for reward without external assistance. During shaping, rats learned to alternate between the starting area and the choice platform. This was accomplished by placing Froot Loops in the choice wells, placing the animals at the start area, and allowing them to alternate between the two for reward. This process was repeated until a rat could achieve 32 alterations in 20 min (Burke et al., 2018).

### Standard Object Discrimination and LEGO^®^ Block Object Discrimination

In the first discrimination task, all rats were given the option of choosing between two distinct objects (Bunny–Rooster) that had variations in size, shape, color, and texture. Half of the rats were rewarded for selecting the bunny and the other half for the rooster. Similar to the previous stages of training, rats were placed in the starting area, ran down the track, and would displace one of the objects, where the correct object and corresponding food well were randomly alternated during the course of the experiment. Upon a correct choice between the objects, the rats were allowed access to the food reward both at the food well and at the starting area, so as to start the next trial, whereas incorrect responses resulted in removal of the Froot Loops piece and no food reward in the well (Burke et al., 2018). This session continued until rats completed 32 trials, and this stage of testing occurred daily until rats reached criterion performance of choosing the correct object 26 out of 32 trials (81.25% correct). To prevent rats from choosing an object solely based on the scent of the object, each object was cleaned thoroughly with 70% ethanol and was rotated out with other exact copies.

Once rats had reached criterion on the standard task, they were then moved onto the LEGO block object Discrimination Task. In this task, rats were given objects constructed out of LEGO blocks and had to discriminate between LEGO objects with similar volume and size (see Figure 1B). The front face of the LEGO objects, that which was visible to rats in the testing space, varied in composition. Methods used to train the rats to discriminate between the two LEGO objects were identical to the methods for classic object discrimination. Training continued until a rat was able to reach the criteria of 81.25% correct (26 correct of 32 trials). Once they had reached criterion performance, animals were trained to discriminate a different novel LEGO object pair in which the correct object was the target for the LEGO block Mnemonic Discrimination task. Once the animals had reached criterion performance of 26 correct of 32 trials, for a final time, rats underwent surgical transection of the perforant path.

### Perforant Path Transection and Cholera Toxin Subunit B Injection

**FIGURE 2:**
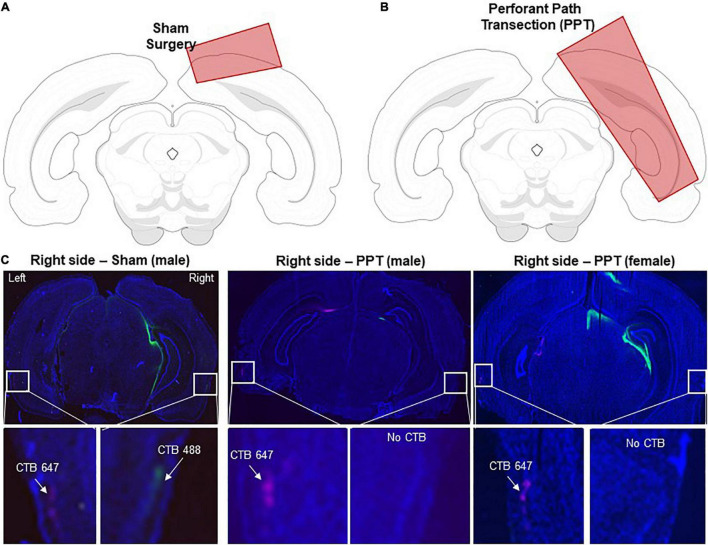
Verification of right hemisphere perforant path transection (Reproduced from Burke et al., 2018). **(A)** A schematic showing the cortical knife cut in the sham surgical procedures. Note, that the retrosplenial and primary visual cortices were sliced. **(B)** A schematic showing the cortical knife cut that was used to transect the right perforant path. **(C)** All rats received injections of the retrograde tracer CTB647 (pink) in the left hippocampus and CTB488 (green) in the right hippocampus. Sham animals had CTB-labeled neurons in the both hemispheres of the lateral entorhinal cortex (left panels). Rats that received the transection (PPT), only had CTB647-labeled cells in the left hemisphere. Nuclei are labeled with DAPI (blue).

Each rat underwent stereotaxic surgery under isoflurane anesthesia (1–3%) to either unilaterally cut the perforant path fibers in the right hemisphere (note, small portion of retrosplenial and primary visual cortex were also cut) or a sham surgery in which only the right hemisphere retrosplenial and primary visual cortices were cut, but fibers were left intact. All animals were injected with the retrograde tracer Cholera Toxin Subunit B in both the left (Alexa Fluor 647 Conjugate, C34778, Invitrogen, Carlsbad, CA, United States) and right (Alexa Fluor 488 Conjugate, catalog #: C22841, Invitrogen, Carlsbad, CA, United States) hippocampus. Cholera Toxin Subunit B conjugated with Alexa Fluor 488 was injected into the right hemisphere to verify the knife cut in animals that received the transection and intact fibers in sham animals. CTB with the Alexa Fluor 647 conjugate was used as a positive control to ensure that the tracer was visible in the entorhinal cortex of the non-lesioned hemisphere. During surgery, an incision was made to expose Bregma and Lambda. A 2.5 mm wide craniotomy was drilled in the skull centered at 1.7 mm anterior to the intra-aural line in males and extending laterally in the right hemisphere from 0.5 to 5.0 mm lateral to the midline. For females, to adjust for differences in skull and brain size, coordinates were modified based on the distance between bregma and the intra-aural line (Paxinosy et al., 1985). In female rats, the craniotomy was centered at 1.9 mm anterior to the intra-aural line and extending from 0.5 to 4.75 mm lateral to the midline. Knife cuts were made using a Micro Feather Ophthalmic Scalpel No. 715 (Electron Microscopy Sciences) which had a triangular blade 7 mm long, 2 mm wide at the base and 0.2 mm thick, with an edge angled 15° to the handle. The knife was mounted on a stereotactic manipulator angled 20° from vertical in the coronal plane, so that the knife edge was angled a total of 35° from vertical. It was positioned at the correct AP coordinate (males: 4.0 mm lateral to the midline on the right side, and then inserted 5.0 mm penetrating at a 20° angle; females: 3.8 mm lateral to the midline on the right side and then inserted 4.75 mm penetrating at a 20° angle). It was then moved 2.5 mm (males) or 2.3 mm (females) medially as measured by the ‘horizontal’ scale of the manipulator, then withdrawn using the ‘vertical’ adjustment of the manipulator. The sham condition followed the same procedure as above except the blade was only inserted 1.0 mm into brain to cut only into the cortex, sparing perforant path fibers. The cortical areas damaged by the sham procedure were the retrosplenial and primary visual cortices, as confirmed by a rat brain atlas (Paxinos and Watson, 2005). All animals performed well on the easier discrimination pairings, and thus the damage to these cortical areas alone did not appear to affect behavioral results. Damaged regions for both sham and transection procedures are shown in Figures 2A,B.

Immediately following the perforant path fiber transection, two additional craniotomies were drilled over the left and right hippocampi for CTB infusions. Placement of two different Alexa Fluor conjugates of this retrograde tracer (488 and 647) enabled verification of the right hemisphere perforant path fiber transection (488) with intact fiber verification in the left hemisphere (647) within the same animals. Each CTB conjugate was diluted to 1% in PBS (Conte et al., 2009a,b). All CTB infusions were made using a Nanoject II Auto-Nanoliter Injector (Drummond Scientific Company) fit with a glass pipette backfilled with the appropriate CTB conjugate. In male rats the CTB was infused at –3.8 mm posterior to Bregma, ± 2.2 mm mediolateral and between –3.4 and –2.1 ventral to the dural surface. The glass pipette was lowered to –3.4 ventral to the dural surface and 50.6 nL of CTB was infused. The pipette was left in place for 1 min and then moved up 100 μm in which another 50.6 nl infusion was placed. This process was repeated at every 100 μm until a final infusion was made at –2.1 mm ventral to dura for a total of 0.708 μL of CTB. This way the tracer infusion included the CA3 and dentate gyrus subregions. Following the final infusion, the pipette was left in place for 150 s, and then slowly advanced up by 0.5 mm where it was left in place for another 150 s allowing for the tracer to diffuse away. After the final waiting period, the pipette was slowly removed from the brain. These infusion procedures were then repeated with other CTB conjugate in the contralateral hemisphere. The CTB infusions in the female rats were identical to those used for the males except the coordinates were adjusted to –3.6 mm posterior to bregma, ±2.1 mm mediolateral and –3.3 to –2.0 mm ventral to the dura. After rats completed all experimental procedures, the perforant path transection was histologically verified. Through the full extent of the hippocampus (from approximately –2 mm to –7 mm posterior to bregma), 20 μm thick sections were sampled approximately every 200 μm, slide mounted, and then stained with the nuclear counterstain DAPI. All SHAM rats had visible CTB in both hemispheres. Rats in the perforant path transection group had CTB647 in the left entorhinal cortex, but no CTB488 was detected in the right hemisphere. Representative images of both hemispheres for male and female transected animals as well as a male sham are shown in Figure 2C.

### Discrimination Retraining and Rodent Mnemonic Similarity Task Testing

After surgery, rats were given 1 week to recover prior to being retrained to distinguish between two distinct LEGO objects until they reached 81.25% correct. Rats then participated in the rodent variant of the mnemonic discrimination task, which tests the target-lure discrimination ability across levels of feature overlap that vary between 0 and 90% (Johnson et al., 2017). Performance on this version of the rodent mnemonic similarity task confirmed a behavioral deficit due to the right perforant path transection and these data were published previously (Burke et al., 2018). Response bias (that is, the animal selecting an object on one side over another, regardless of identity), response time, and checking behavior were evaluated in Burke et al. (2018), and response bias was not lateralized to the side of the transection or sham lesions. The behavioral data presented in the current paper (Figure 1C).

### Compartment Analysis of Temporal Activity With Fluorescence *in situ* Hybridization (catFISH)

On the final day of behavioral testing rats performed two 5 min behavioral epochs of LEGO block object discrimination, separated by 20 min in which they were returned to the home cage. During one epoch, rats performed the 70% overlap discrimination and in the other epoch rats were tested on the 90% overlap condition, with the order of the different discrimination problems counterbalanced between animals. Due to the two-epoch design on the experiment day, the 50% overlap lure (Lure 1) was not included. See Figure 1B for an image of the different stimuli that were used in these two overlap conditions. Immediately following completion of the second behavioral epoch, rats were placed into a glass bell jar saturated with isoflurane vapor and were rapidly anesthetized prior to being decapitated with a rodent guillotine. Immediately following decapitation, rat brains were extracted, and flash frozen in chilled isopentane (–50°C). Brains were then stored in a freezer at –80°C until cryosectioning. Twenty-micron thick coronal sections of both hemispheres were cut on a cryostat and arranged on Superfrost Plus slides (Fisher Scientific; Hampton, NH, United States) with whole brains from three experimental animals that always included at least one transection and one sham animal. Additionally, a hemisected cage control and a MECS (maximal electroconvulsive shock administered prior to sacrifice as a positive control) animal were included to ensure that slight deviations in staining between slides would not confound the data. The MECS condition served as a positive control to ensure that the *in situ* hybridization worked. This was verified by qualitative examination, and these data were not quantitively analyzed. Tissue was then stored at –80°C until it could be processed using fluorescence *in situ* hybridization (FISH). Every 10th slide was stained with the nuclear counterstain DAPI to examine for projection neurons in the lateral entorhinal cortex (LEC). This procedure has been confirmed to be a valid method by which to disconnect the perforant pathway as published previously in Burke et al. (2018).

Twenty-four hours before FISH occurred, brain tissue was moved to a –20°C freezer to prepare for thawing the subsequent day. FISH was performed as previously described (Guzowski et al., 1999; Burke et al., 2005, 2012). Briefly, a commercial transcription kit and RNA labeling mix (Ambion REF #: 11277073910, Lot #: 10030660; Austin, TX, United States) was used to generate a digoxigenin-labeled riboprobe with a plasmid template containing a 3.0 kb *Arc* cDNA (generously provided by Dr. A Vazdarjanova; Augusta University). Tissue was incubated with the probe overnight and *Arc* positive cells were detected with anti-digoxigenin-HRP conjugate (Roche Applied Science Ref #: 11207733910, Lot #: 14983100; Penzberg, Germany). Cyanine-3 (Cy3 Direct FISH; PerkinElmer Life Sciences, Waltham, MA, United States) was used to visualize labeled cells and nuclei were counterstained with DAPI (ThermoFisher Scientific).

### Imaging and Analysis

Optical z-stacks were acquired with a Keyence BZ-X7000 all-in-one fluorescence microscope and acquired with the Keyence software (Keyence; Osaka, Osaka Prefecture, Japan). Slides were loaded into the Keyence and were clasped in place. The brain sections were then initially viewed under 2X zoom to verify anatomical location of regions of interest. For the LEC, the rhinal sulcus was used a landmark and images were acquired from ventral to the sulcus once the prominent fan cells of layer II were visible. These clearly distinguished entorhinal cortex from area 35 of the perirhinal cortex (Burwell, 2001). Lateral entorhinal cortical Images were acquired from approximately –4.8 to –6.0 mm posterior to bregma. For CA3, images were acquired from dorsal CA3 between approximately –3.5 and –4.2 mm posterior to bregma. Analyses of *Arc* expression are focused on dorsal CA3 because a previous inactivation study showed that muscimol infusions into dorsal hippocampus impaired performance on the mnemonic discrimination task (Johnson et al., 2019). Moreover, neurophysiological recordings that have revealed hyperactivity in CA3 of aged rats have been obtained from primarily dorsal regions (Wilson et al., 2005; Robitsek et al., 2015).

**FIGURE 3:**
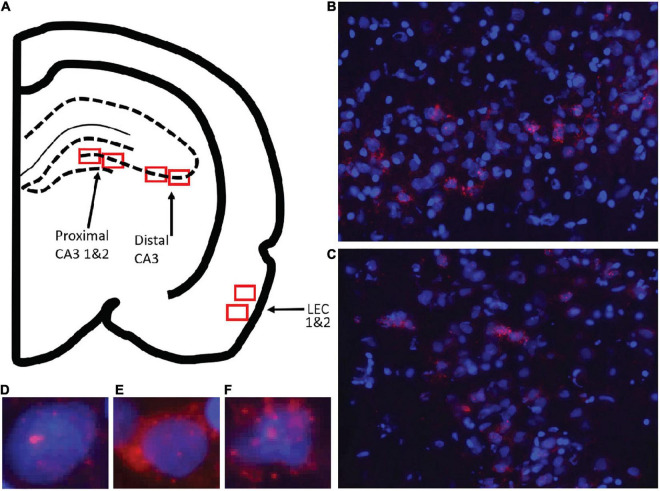
Images taken for analysis of immediate early gene *Arc* expression in CA3 (proximal, distal) and lateral entorhinal cortex (LEC). **(A)** Schematic of the locations for image collection. Two areas were sampled from each region of interest shown by the red boxes. **(B)** Example image of CA3 and **(C)** LEC analyzed for *Arc* expression. Overall cell count was determined, and cells were then classified as being positive for *Arc* in the nucleus or foci **(D)**, cytoplasm **(E)**, or both foci and cytoplasm **(F)**.

The Keyence objective was then switched to 40× zoom to acquire optical z-stacks at increments of 1 μm. The cell bodies were first viewed under the DAPI channel and exposure was adjusted so that neurons and glia could be differentiated. Additionally, the *Arc* channel was turned on and exposure was adjusted to minimize background while retaining cytoplasmic labeling. For both the cell body (DAPI) and *Arc* channels (Cy3) channels exposure, gain and offset was kept consistent across images within a single slide. Images were taken in both the left and right hemispheres in the areas of interest from 2 to 3 slides for each animal. For several rats only two slides had usable tissue that was not damaged. In the hippocampus, two images were taken from each slide for distal CA3 (area within the hilus of the dentate gyrus), and proximal CA3 (closer to CA2), so that the two images did not overlap. There was an average of 59 cells in each CA3 image. In the LEC, a dorsal image (closer to the rhinal sulcus) and a more ventral image were taken. There was an average of 63 cells in each LEC image. Figure 3 shows the anatomical regions that images were acquired from. Analyses were focused on the lateral subdivision of the entorhinal cortex, and images were not acquired from the medial entorhinal cortex (MEC). This is because activity in the LEC is modulated by objects (Deshmukh and Knierim, 2011), and lesions of this region impair associations that involve objects (Wilson et al., 2013a,b). An explicit involvement of the MEC in object discrimination has not been reported, and this region appears to be more involved in path integration and spatial navigation (Hafting et al., 2005; Steffenach et al., 2005; Knierim et al., 2014). Additionally, the MEC was difficult to identify in the coronal brain sections used for all *in situ* hybridization analyses.

Prior to counting, image file names were coded so that experimenters were blind to condition. Initially, the *Arc* channel was turned off to prevent bias while identifying nuclei. Only neurons that were completely contained within the image stack were counted. These neurons were identified by only counting nuclei that were visible in the Median 20% of the z-stack. Any neurons that were cut off by the edges were also excluded. The *Arc* channel was then turned on and cells were classified as being positive for *Arc* in the nucleus (foci+), in the cytosol (cyto+), in both the nucleus and cytosol (both), or no expression (neg). In order to be considered “foci+”, foci visible in at least 4 adjacent planes. A “cyto+” cell was defined as a cell with *Arc* following the outer contours of at least 30% of the nucleus. A “both” cell was defined as a cell that met the criteria for foci+ and cyto+. A “neg” cell was defined as a cell that did not meet any of these criteria.

### Statistical Analyses

Analyses were performed with R Studio and SPSS for Windows. Behavioral and *Arc* expression variables were tested for statistical significance with repeated measures ANOVAs with similarity of object pairs as the within-subjects factor and PPT or sham surgery, brain hemisphere, and sex as the between-subjects factors. Potential relationships between behavior and *Arc* expression variable were explored with principal components analysis.

## Results

### Mnemonic Similarity Discrimination Testing

Rats performed two behavioral epochs of LEGO block object discrimination testing, counterbalanced between 70 and 90% visual overlap target-lure LEGO object pairs. Figure 1C shows the mean percent correct for the Sham and perforant path transection (Cut) groups in the different overlap conditions. Mean performance values for each trial type were entered into a repeated measures ANOVA with lure similarity (70% versus 90%) as a within-subjects factor and sex and experimental surgical group (Sham or Cut) as between-subjects factors. There was a main effect of lure similarity (*F*_[1,28]_ = 62.361, *p* < 0.001), and overall rats averaged 90.9% (±1.41 SEM) correct on the 70% similarity trials and 65.2% (±2.83 SEM) correct on 90% similarity trials. Furthermore, there was a main effect of experimental surgical condition (*F*_[1,28]_ = 6.072, *p* = 0.020), with Sham animals having more correct trials compared to the Cut rats. Performance did not vary by sex (*F*_[1,28]_ = 0.752, *p* = 0.393).

### Arc Expression in Relation to Behavioral Epochs

**FIGURE 4:**
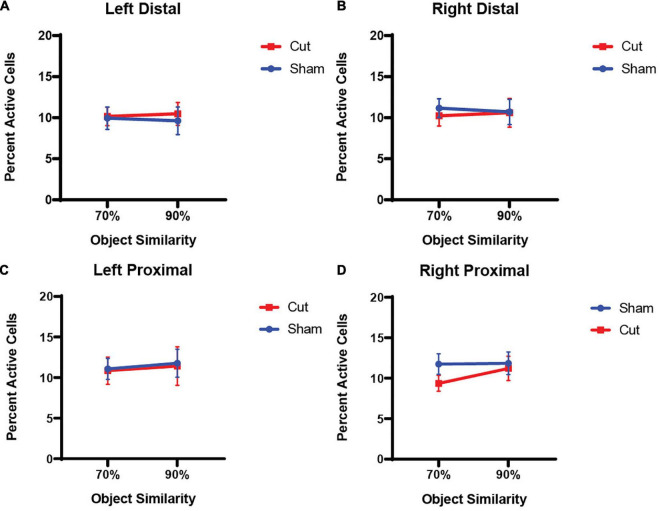
Percentage of active cells, as determined by expression of the immediate early gene *Arc*, in the CA3 subregion of the hippocampus (n’s: Sham = 12♀/7♂; Cut = 12♀/7♂). There were no within- or between-subjects effects found for either distal **(A,B)** or proximal **(C,D)** CA3.

Animals that remained in their home cage for the duration of the experiment and performed no behavioral assessments showed low *Arc* expression (Total Activity in the LEC: 5.81%; CA3 distal: 3.87%; CA3 proximal: 4.17%). Figures 4A–D show the average population activity between the different overlap epochs inferred from *Arc* expression in proximal and distal regions of CA3 in both the left (Intact) and right (Cut) hemispheres (see Figure 3A for location of proximal and distal regions). A repeated measures ANOVA revealed no main effect of Object Similarity (*F*_[1,55]_ = 0.3435, *p* = 0.560), Surgical Condition (*F*_[1,55]_ = 0.5277, *p* = 0.471), Hemisphere (*F*_[1,55]_ = 0.0252, *p* = 0.875), or Sex (*F*_[1,55]_ = 1.5889, *p* = 0.213) on CA3 *Arc* expression. Furthermore, there were no interaction effects between any of the between- or within-subjects factors (*p* > 0.364 for all comparisons).

**FIGURE 5:**
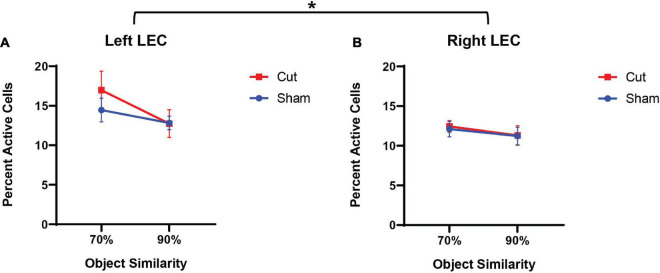
Percentage of active cells, as determined by expression of the immediate early gene *Arc*, in the Left **(A)** and Right **(B)** Lateral Entorhinal Cortex (n’s: Sham = 12♀/7♂; Cut = 12♀/7♂). Quantified *Arc* expression in the left LEC was higher than the right hemisphere (*F*_[1,51]_ = 4.283, *p* = 0.044), but there was no effect of surgical condition or sex. Activity was lower in both hemispheres and across surgical conditions for 90% similarity trials compared to 70% similarity (*F*_[1,55]_ = 5.8482, *p* = 0.019). * indicates a significant effect (*p* ≤ 0.05).

Figure 5 shows the average population activity in the left (A) and right (B) hemispheres of the LEC. A repeated measures ANOVA showed a main effect of the within-subjects factor of Object Similarity (*F*_[1,55]_ = 5.8482, *p* = 0.019) and between-subjects factor of Hemisphere (*F*_[1,55]_ = 4.28338, *p* = 0.044), but no effect of surgical Condition (Sham versus Cut: *F*_[1,55]_ = 0.04222, *p* = 0.838) or Sex (*F*_[1,55]_ = 0.00322, *p* = 0.955). The significant between-subjects effect of hemisphere indicated that overall population activity was higher in the left hemisphere of the LEC in all animals compared to the right. This observation of activity differences between the left and right LEC suggests that the cortical damage induced by the surgical procedures in both the Sham and Cut conditions led to reduced LEC activity. Furthermore, in both surgical groups, *Arc* expression during the 90% overlap discrimination condition was lower compared to 70% feature overlap condition (*F*_[1,55]_ = 5.8482, *p* = 0.019). This could be due to the more unique features of the LEGO objects in the 70% condition resulting in more neuron activation, in contrast to the 90% condition in which the two objects are more likely to be perceived as identical.

### Similarity Scores

**FIGURE 6:**
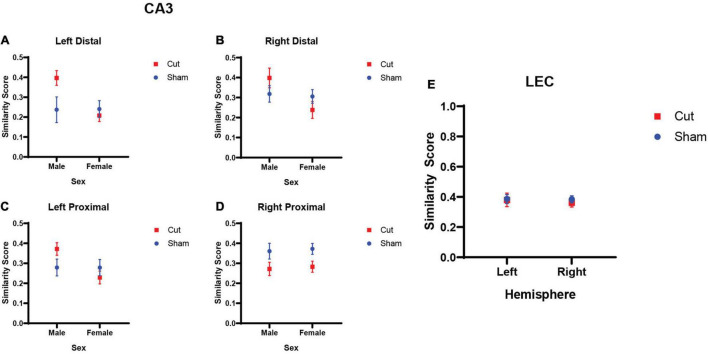
Similarity score of population activity between epochs 1 and 2 as evaluated by *Arc* expression. **(A–D)** CA3 similarity scores did not show a main effect of hemisphere (n’s: Sham = 12♀/7♂; Cut = 12♀/7♂). There was a main effect of sex (*F*_[1,27]_ = 6.1465, *p* = 0.020), and an interaction effect between sex and surgical condition (*F*_[1,27]_ = 4.5518, *p* = 0.042). **(E)** LEC similarity scores showed no main effect of hemisphere (*F*_[1,25]_ = 0.903, *p* = 0.351), sex (*F*_[1,25]_ = 0.139, *p* = 0.712), or surgical condition (*F*_[1,51]_ = 0.057, *p* = 0.813).

In order to investigate the degree to which population activity overlapped across the 70 and 90% overlap behavioral epochs, while accounting for differences in the size of neuronal ensembles that were positive for *Arc* mRNA, similarity scores were calculated. The similarity scores between epochs 1 and 2 were calculated as:


$$\frac{p\,B-\left(p\,E\,1\,x\,p\,E\,2\right)}{\frac{p\,E\,1\,x\,p\,E\,2}{2}-\left(p\,E\,1\,x\,p\,E\,2\right)}$$

where, pB represents the proportion of cells active during both epochs, pE1 the proportion of cells active during epoch 1, and pE2 those active during epoch 2. Figures 6A–D show the similarity scores across proximal and distal CA3 in both the left and right hemispheres. A repeated measures ANOVA revealed that males had significantly higher similarity scores than females (*F*_[1,27]_ = 6.1465, *p* = 0.020). Furthermore, there was not a significant interaction between condition and hemisphere (*F*_[1,27]_ = 2.926, *p* = 0.099), but the interaction between sex and surgical condition did reach statistical significance (*F*_[1,27]_ = 4.5518, *p* = 0.042). The reasons for these sex differences will require additional exploration. Figure 6E shows the similarity score in the left and right hemispheres of the LEC across surgical conditions. A repeated measures ANOVA found no main effect of or interaction effects between the factors of Hemisphere, Condition, or Sex on LEC similarity scores (*p* ≥ 0.351 for all comparisons).

### Relationship Between Arc Expression Patterns and Behavioral Performance

To interrogate whether there was a potential relationship between the expression patterns of the immediate early gene *Arc* in any of the hippocampus regions of interest and discrimination performance, data reduction was performed with a principal component analysis (PCA) that included all measures of *Arc* expression. A PCA with varimax rotation was performed due to split loadings; however, one variable was still shown to have cross-loadings for components 1 and 3, thus was dropped and the PCA was run again. According to the total variance explained (see Table 1), the model using the top six components (eigenvalues >1.0) explained 77.613% of variance in the data. The first component (eigenvalue = 3.670) corresponded to activity during the 70% object similarity trials and accounted for 21.590% of variance in the data. Increased *Arc* transcription in both left and right hemisphere distal and proximal CA3 regions positively loaded onto the first component (≥0.660), and similarity score for the right LEC negatively loaded onto component 1 (–0.606). The second component (eigenvalue = 2.777) corresponded to activity during the 90% object similarity trials and uniquely accounted for 16.333% of variance in the data. Increased *Arc* transcription in both left and right hemisphere for distal CA3 as well as right proximal CA3 regions positively loaded onto the second component (≥0.584). The third component (eigenvalue = 2.215) corresponded to left hemisphere CA3 similarity scores and accounted for 13.027% of variance. Higher similarity scores (values approaching 1) for both proximal and distal CA3 subregions positively loaded onto component 3 (≥0.793). *Arc* expression in the LEC during both overlap conditions loaded positively (≥0.730) onto the 4th component and accounted for 11.021% of the variance. CA3 similarity score negatively loaded onto the component (–0.644). The variables that loaded onto the 5th and 6th components did not have an obvious relationship to each other and accounted for less than 10% of the variance.

**TABLE 1:** Rotated component matrix with varimax rotation from principal component analysis (PCA).

PCA						
Component	1	2	3	4	5	6
Eigenvalue; % variance	3.670; 21.590%	2.777; 16.333%	2.215; 13.027%	1.874; 11.021%	1.477; 8.689%	1.182; 6.953%
Left proximal CA3 70%	0.851					
Right distal CA3 70%	0.747					
Left distal CA3 70%	0.718					
Right proximal CA3 70%	0.660					
Right lateral entorhinal cortex (LEC) similarity score	–0.606					
Right proximal CA3 90%		0.874				
Right distal CA3 90%		0.858				
Left distal CA3 90%		0.584				
Left distal CA3 similarity score			0.917			
Left proximal CA3 similarity score			0.793			
Left LEC 70%				0.794		
Left LEC 90%				0.730		
Right distal CA3 similarity score				–0.644		
Right proximal CA3 similarity score					–0.875	
Left LEC similarity score						–0.788
Right LEC 90%					0.533	0.547
Right LEC 70%						

**FIGURE 7:**
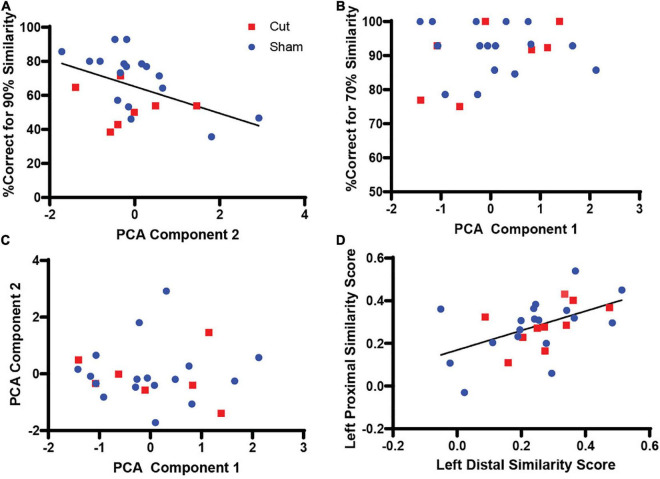
Correlations between regression scores from PCA analysis and corresponding behavioral epoch performance (n’s: Sham = 12♀/7♂; Cut = 12♀/7♂). **(A)** Comparison of PCA Components 1 and 2, which correspond to Arc expression during the 70 and 90% similarity trials, respectively (Pearson’s *r* = 0.0, *p* = 1.0). **(B)** Comparison of PCA Component 1 and percent correct trials during 70% similarity trials yielded no significant correlation (Pearson’s *r* = 0.141, *p* = 0.512). **(C)** Comparison of PCA Component 2 and percent correct trials during 90% similarity trials showed a significant correlation (Pearson’s *r* = –0.463, *p* = 0.023). **(D)** Comparison of left distal and proximal CA3 similarity scores showed a significant correlation (Pearson’s *r* = 0.512, *p* = 0.004).

To evaluate any relation between *Arc* activity and behavioral performance, a two-tailed Pearson’s Correlation score was calculated between the regression factors for components 1 and 2 and the corresponding behavioral epoch performance (Table 2, Figure 7). The test between the first factor, corresponding with *Arc* activity during 70% object similarity trials, and animals’ percent correct trials during the 70% overlap epoch did not show a correlation between *Arc* expression and performance (Pearson’s *r* = 0.141, *p* = 0.512). Interestingly, the same analysis of the second regression factor from the PCA, which corresponded to activity during the 90% object similarity trials, and animals’ percent correct trials during the 90% overlap epoch revealed a significant negative correlation between *Arc* expression and discrimination performance (Pearson’s *r* = –0.463, *p* = 0.023), indicating that animals with more CA3 activity had worse performance on the 90% feature overlap condition of the mnemonic similarity task.

**TABLE 2:** Pearson’s Correlations between percent of correct trials during 70 or 90% overlap conditions and either **(A)** the regression factor score for PCA Component 1 or **(B)** regression factor score for PCA Component 2.

Pearson’s Correlations				
**A**		**Percent correct 70% similarity**	**Percent correct 90% similarity**	**PCA component 1**

Percent correct 70% similarity	Pearson correlation	1	0.000	0.141
	Significance (2-tailed)		0.999	0.521
	*N*	32	32	24
Percent correct 90% similarity	Pearson correlation	0.000	1	0.053
	Significance (2-tailed)	0.999		0.805
	*N*	32	32	24
PCA component 1	Pearson correlation	0.141	0.053	1
	Significance (2-tailed)	0.512	0.805	
	*N*	24	24	24

**B**		**Percent correct 70% similarity**	**Percent correct 90% similarity**	**PCA component 2**

Percent correct 70% similarity	Pearson correlation	1	0.000	0.067
	Significance (2-tailed)		0.999	0.757
	*N*	32	32	24
Percent correct 90% similarity	Pearson correlation	0.000	1	–0.463*
	Significance (2-tailed)	0.999		0.023
	N	32	32	24
PCA Component 2	Pearson correlation	0.067	–0.463*	1
	Significance (2-tailed)	0.757	0.023	
	N	24	24	24

*Indicates significant effect.

## Discussion

This study tested the hypothesis that perforant path fiber damage leads to increased neuronal activity in CA3, which has been reported in aged animals (Wilson et al., 2005; Yassa et al., 2011b; Robitsek et al., 2015; Thomé et al., 2016; Lee H. et al., 2021) and older adults (Yassa et al., 2011a,b; Reagh et al., 2018). A previous behavioral study with these same animals, reported that unilateral damage to the perforant path impaired the ability to discriminate between a target and 90% similar lure (Burke et al., 2018). Although the experimental procedures were different between the current study and Burke et al. (2018), the effect unilateral perforant path damage persisted. During the experimental procedures of the current study, all animals were able to correctly discriminate between a target object and 70% similar lure object significantly more than when the lure had 90% feature overlap with the target object. Moreover, there was a significant difference in performance accuracy based upon surgical condition, with animals that received perforant path transection making more errors than the animals that underwent sham surgery (Figure 1C). While the fiber transection resulted in behavioral impairments, there were no significant effects of surgical condition on *Arc* expression in CA3 or the LEC. Additionally, there were no sex differences in behavioral performance or percent of active cells during either the 70 or 90% overlap conditions. Together these observations suggest that a unilateral loss of perforant fibers does not significantly alter neuronal activation in CA3 and LEC, despite this surgical treatment leading to altered behavioral performance. These observations lend further support to notion that intrinsic circuits of the hippocampus are reliant on local computations and are therefore robust against alterations in afferent input (Brun et al., 2008; Zutshi et al., 2022).

A few limitations need to be mentioned with regards to this conclusion that perforant path fiber damage does not induce CA3 hyperactivity. First, *Arc* expression was only evaluated in dorsal CA3. This focus was based on a previous inactivation study showing that muscimol infusions into dorsal hippocampus impaired performance on the mnemonic discrimination task (Johnson et al., 2019), as well as neurophysiology data that reported hyperactivity in dorsal CA3 of aged rats (Wilson et al., 2005; Robitsek et al., 2015). Moreover, it has not been established if more ventral areas of CA3 are also involved in mnemonic discrimination task performance. However, it has been previously reported that there is a higher proportion of cells that express *Arc* in more ventral areas of CA3 in aged behaviorally impaired rats compared to young animals and rats without impairment (Maurer et al., 2017). Because more ventral regions have fewer cells that express Arc, it is conceivable that there is more parametric space in ventral CA3 to detect an increase. Thus, it will be important to include this analysis in future studies. Second, while perforant path fiber damage in young animals did not recapitulate dorsal CA3 hyperactivity, despite leading to behavioral impairments that have been linked to this aberrant activity (Yassa et al., 2011a,b; Maurer et al., 2017), it is still possible that age-related fiber degradation is the impetus of CA3 hyperactivity in old animals. There are many cellular changes within the aged CA3 circuit that include disrupted phenotypic expression profiles of interneurons (Cadacio et al., 2003; Koh et al., 2010; Spiegel et al., 2013), alterations in transcription (Haberman et al., 2011), and a redistribution of synaptic weights (Buss et al., 2021). All these factors could affect the intrinsic excitability of CA3 pyramidal cells (Simkin et al., 2015). It is conceivable that this constellation of age-related changes in the CA3 circuit is then unable to compensate in the face of perforant path fiber damage that then ultimately manifests of hyperactivity. In young animals, the fiber damage that was experimentally induced here may not disturb the circuit because compensation is provoked, perhaps from commissural CA3 projections or intrinsic mechanisms, that ultimately maintain normal inhibitory/excitatory balance when afferent input is disrupted.

When the data from both surgical groups were analyzed together, *Arc* activation was related to participation in the behavioral task. Specifically, in both hemispheres of the LEC there was more *Arc* expression associated with the 70% overlap condition of the mnemonic similarity task compared to the 90% overlap condition. There are at least two potential explanations for this observation. First, rats received more reward in the 70% overlap condition compared to when the feature overlap was 90%. It is conceivable to more reward resulted in more LEC activation. In support of this idea, the LEC receives direct innervation from dopamine neurons in the ventral tegmental area (Loughlin and Fallon, 1984; Oades and Halliday, 1987; Björklund and Dunnett, 2007). Furthermore, a recent study reported that neurons in the LEC were active during the acquisition of cue-reward association (Lee J. Y. et al., 2021). An alternative, but not mutually exclusive possibility is that in the 70% overlap condition, the two objects are more likely to be perceived as distinct, while the 90% overlap objects are more likely to be perceived as identical. By this account, two unique objects could produce more LEC activation then two identical objects, which have fewer unique features. Previous studies have reported that LEC neurons recorded from rats are responsive to objects (Deshmukh and Knierim, 2008, 2011; Tsao et al., 2013; Knierim et al., 2014), which supports the notion that when an animal experiences more unique objects, a larger proportion of LEC neurons will be active. In contrast to the LEC, the proportion of neurons in CA3 that were positive for *Arc* mRNA did not differ between the 70 and 90% feature overlap conditions.

Similarity score was used to quantify the extent that transected versus sham animals showed differences in the population overlap of active neuronal ensembles between the different overlap conditions. This measure accounts for differences in overall activation and the expected overlap based on chance (Vazdarjanova and Guzowski, 2004). Scores range from 0 to 1, with a higher similarity score indicating greater population overlap in cells active between behavioral epochs. For the LEC, the similarity of population activity between the 70% and 90% overlap condition was around 0.4 for all conditions and did not differ by hemisphere, sex, or surgical condition. For CA3, there were differences in the similarity score by surgical condition that interacted with sex. For left proximal, left distal and right distal subregions of CA3, similarity score was higher for male compared to female rats, but this effect was only evident in the rats with right perforant path transection. The reason for the sex difference is not apparent and requires further investigation, but it could be due to the transection affecting males more than females, which has been reported for ventral hippocampal lesions (Beninger et al., 2009). In the right proximal CA3 subregion, the similarity score was lower in the rats that received perforant path transection. As this area of CA3 does not receive direct entorhinal cortical input (Witter et al., 2000), the reason for this difference is not evident.

Dimension reduction was performed to examine what *Arc* expression variables were related to each other. The proportion of neurons expressing *Arc* during the 70% overlap condition in all subregions of CA3 positively loaded onto first principal component. This is consistent with the anatomy of CA3 that contains strong auto-associative projections (Amaral and Witter, 1995; Witter, 2007), which could support similar activity patterns among different neurons in the CA3 circuit. The first principal component did not correlate with performance during the 70% overlap condition, which could be due to the near ceiling level of performance during this condition that did not allow for sufficient parametric space to detect neural-behavioral relationships. The second principal component was positively associated with the proportion of CA3 neurons that expressed *Arc* during the 90% overlap condition in right distal and proximal CA3, as well as left distal CA3. Importantly, the second principal component positively correlated with performance on the 90% overlap condition of the mnemonic similarity task. This observation suggests that more activity in CA3 supports the ability to discriminate between stimuli that share features. Importantly, total activation in CA3 was between 8 and12% for all animals. If these data are considered within the context of previous research showing worse performance on the mnemonic similarity task with increased CA3 activity (Yassa et al., 2010; Maurer et al., 2017), it suggests that there is an inverted U-shaped relationship between CA3 activity and the ability to discriminate between stimuli that share features. Discrimination accuracy will wane if CA3 activity is too low or two high (Kent et al., 2016).

Notably the present data call into question models of pattern separation and completion (Marr, 1971; Rolls, 2013). These models predict that computations within the dentate gyrus serve to orthogonalize activity between two overlapping experiences represented in the cortex. This model would predict that activity overlap in CA3 would be reduced compared to the LEC, as the dentate gyrus would have separated the overlapping input patterns between the two epochs of behavior. In the current experiment, we did not observe large differences in orthogonalization of population activity overlap between the LEC and CA3, as indicated by the similarity scores. While it is conceivable that the dentate gyrus would have shown reduced overlap, it is unclear as to why input patterns would be orthogonalized by the dentate gyrus to then become more similar in CA3. Pattern separation and completion network models assume that patterns of brain activity represent unique states and that the hippocampus generates sharp transitions in activity between two well-learned states as input gradually changes (McNaughton and Morris, 1987; Guzowski et al., 2004; Leutgeb et al., 2005). According to this theory, the hippocampus makes binary decisions, but would be incapable of reconciling situations in which small changes must be tracked and incorporated within a continuous context (Maurer and Nadel, 2021). To solve the mnemonic similarity task, animals need to compare a current stimulus to a learned target to determine if it is identical, while simultaneously detecting small differences in the configuration of features to detect and ignore the lure object. This task cannot be adequately solved with a single binary pattern completion or separation computation, but rather is more likely to be supported by re-entrant loops, and amorphous networks that can monitor stimulus equivalence while tracking perceptual continuities and discontinuities (Maurer and Nadel, 2021).

Overall, this project shows that a unilateral transection of the perforant path does not recapitulate age-related changes in neural activity as measured by immediate early gene *Arc* expression. This suggests that while perforant path degradation is a feature of aging and may contribute to age-related cognitive decline, it does not seem to be the catalyst for age-related changes in CA3 activity patterns. These results therefore beg the question of whether perforant path dysfunction is more easily compensated for in younger animals or if other factors common to advanced age compound with perforant path degradation in a unique way to cause cognitive deficits seen in older populations. To further untangle the processes involved in neural compensation and cognitive resilience, it will be important to examine if there are effects of a perforant path transection on other regions known to be involved in object recognition such as the perirhinal cortex or CA1 region of the hippocampus. If these regions are found to have higher levels of activation as measured by *Arc* expression, it could indicate a compensatory effect that mitigates any disruption of cognitive function due to loss of perforant path fibers from the LEC.

## Data Availability Statement

The raw data supporting the conclusions of this article will be made available by the authors, without undue reservation.

## Ethics Statement

The animal study was reviewed and approved by Institutional Animal Care and Use Committee at the University of Florida.

## Author Contributions

TC analyzed the data and wrote and edited the manuscript. ST, KL, JT, and CW collected and analyzed the data. CL created figures and edited the manuscript. SB and AM contributed to the conception and design of the study, mentored trainees, and helped with analysis and writing of the manuscript. All authors contributed to the article and approved the submitted version.

## Conflict of Interest

The authors declare that the research was conducted in the absence of any commercial or financial relationships that could be construed as a potential conflict of interest.

## Publisher’s Note

All claims expressed in this article are solely those of the authors and do not necessarily represent those of their affiliated organizations, or those of the publisher, the editors and the reviewers. Any product that may be evaluated in this article, or claim that may be made by its manufacturer, is not guaranteed or endorsed by the publisher.
